# Robust Classical and Quantum Polarimetry with a Single Nanostructured Metagrating

**DOI:** 10.1021/acsphotonics.3c01287

**Published:** 2024-02-15

**Authors:** Shaun Lung, Kai Wang, Nicolas R. H. Pedersen, Frank Setzpfandt, Andrey A. Sukhorukov

**Affiliations:** † Abbe Center of Photonics, Friedrich-Schiller Universität, Albert-Einstein-Straße 15, Jena 07745, Germany; ‡ ARC Centre of Excellence for Transformative Meta-Optical Systems (TMOS), Department of Electronic Materials Engineering, Research School of Physics, The Australian National University, Canberra, ACT 2600, Australia; § Department of Physics, McGill University, 3600 rue University, Montreal, Quebec H3A 2T8, Canada; ∥ Fraunhofer Institute for Applied Optics and Precision Engineering, Jena 07745, Germany

**Keywords:** metamaterials, nanophotonics and photonic crystals, polarization-selective devices, polarimetry

## Abstract

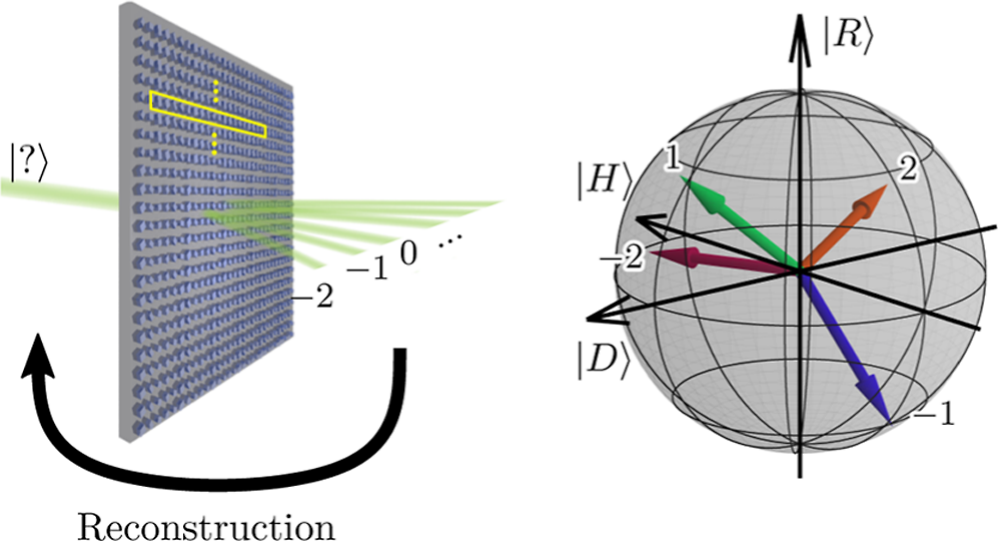

We formulate a new conceptual approach for one-shot complete polarization state measurement with nanostructured metasurfaces applicable to classical light and multiphoton quantum states by drawing on the principles of generalized quantum measurements based on positive operator-valued measures. Accurate polarization reconstruction from a combination of photon counts or correlations from several diffraction orders is robust with respect to even strong fabrication inaccuracies, requiring only a single classical calibration of the metasurface transmission. Furthermore, this approach operates with a single metagrating without interleaving, allowing for a reduction in metasurface size while preserving high transmission efficiency and output beam quality. We theoretically obtained original metasurface designs, fabricated the metasurface from amorphous silicon nanostructures deposited on glass, and experimentally confirmed accurate polarization reconstruction of laser beams. We also anticipate robust operation under changes in environmental conditions, opening new possibilities for space-based imaging and satellite optics.

## Introduction

Single-shot optical polarimetry using ultrathin nanostructured metasurfaces opens up new opportunities for diverse applications,^1−3^ facilitating the measurement of both classical^4−8^ and quantum^9^ polarization states. Whereas traditional polarimetry involves multiple measurements performed via physically varying bulk optical elements such as waveplates and polarizers,^10^ single-shot approaches remove the need for reconfigurability, thereby avoiding the associated measurement errors and facilitating real-time polarization state monitoring^11,12^ that can be combined with spectral imaging.^13−16^

Similar to conventional classical^10^ and quantum polarimetry,^17^ metasurfaces were initially designed to perform several complementary projection measurements,^18^ such that each of the outputs corresponds to a specific polarization.^1−4^ This functionality can be accomplished with metasurfaces that split particular polarization components into distinct diffraction orders or focal spots. For the tomographic characterization of quantum states, metasurfaces were previously developed by interleaving several metagratings whose number scales linearly with the number of photons.^9^ However, the simple interleaving (i) limits the device’s compactness since the photons have to spatially overlap with multiple gratings at once and (ii) introduces output beam distortions that reduce the detection efficiency. This poses a question on how to overcome such limitations and thereby support the emerging applications of metasurfaces in quantum imaging.^19−21^

We reveal that efficient polarimetry with metasurfaces can be accomplished without the commonly considered requirement of realizing close-to-perfect polarization projection measurements. Remarkably, even if each of the metasurface output ports represents a partial polarizer operation that by itself provides inconclusive information about the input state, a tailored combination of all outputs allows for very accurate polarization reconstruction. We achieve this by adopting the framework of generalized quantum measurements based on positive operator-valued measures (POVMs)^22−28^ for the metasurface design. Such an approach fundamentally improves the robustness with respect to nanofabrication inaccuracies and also extends the flexibility in metasurface designs, allowing, in particular, higher efficiency and output beam shaping with small-area metasurfaces. We present simulation results for one- and two-photon states. Then, we experimentally demonstrate the operation with laser light that illustrates the classical regime and also emulates a single-photon quantum case.^29^

## Theory of Multiphoton Polarization Measurements

**Figure 1:**
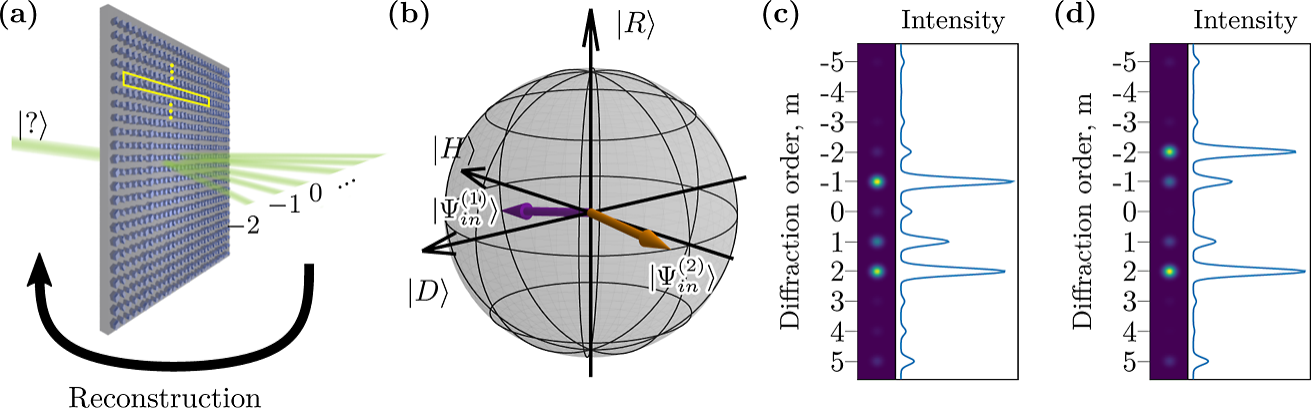
(a) Conceptual sketch of single-metagrating polarimetry. Highlighted in yellow is a single 16 resonator unit cell. From the output diffraction orders, any unknown input state may be reconstructed by knowing the metasurface instrument matrix. (b) Poincaré sphere representation of two arbitrarily chosen input states |Ψ_in_^(1)^⟩ and |Ψ_in_^(2)^⟩ characterized in Jones formalism by the polarization angles and phases (1.5, 0.1) and (0.1, 1.5), respectively. (c,d) Intensities of output diffraction orders corresponding to the respective input states |Ψ_in_^(1)^⟩ and |Ψ_in_^(2)^⟩.

We first formulate a general theory of polarization measurements. We consider a metasurface, which splits an input beam into several diffraction orders, as sketched in Figure 1a. Then, the measurements of photon correlations between the output ports can be used to reconstruct the input quantum state. In a previous study,^9^ it was assumed that the photon polarization was specifically selected at each diffraction order. This effectively required the metasurface to act as a near-perfect polarization splitter, which came at the cost of larger device area requirements due to the need to interleave several gratings and the distortion of the output beam profile. In the following, we show that such limitations can be removed, allowing for a compact and robust metasurface design.

Since we consider a linear regime, the transformation of quantum states can be expressed through the classical Jones transfer matrices **T**^(*m*)^ to each of the diffraction order numbers *m*
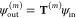
1where ψ_in_ and ψ_out_^(*m*)^ are the classical input and output polarization states in the form of Jones vectors at the respective diffraction orders. Each diffraction order functions, in general, as a partial polarizer acting on the incoming light. It is then convenient for the following analysis to perform a singular value decomposition of the transfer matrices
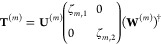
2where ζ_*m*,1_ and ζ_*m*,2_ ≥ 0 are the singular values, **U**^(*m*)^ = [**U**_1_^(*m*)^, **U**_2_^(*m*)^] and **W**^(*m*)^ = [**W**_1_^(*m*)^, **W**_2_^(*m*)^] are unitary matrices, the subscripts indexing the respective columns. We choose the order of singular values, such as ζ_*m*,1_ ≥ ζ_*m*,2_. If ζ_*m*,2_ = 0, the polarization state at the corresponding diffraction order is fixed as for the case of a perfect polarizer. However, in a general case of ζ_*m*,2_ > 0, the metasurface effectively acts as a partial polarizer, with a power extinction ratio of .

We now formulate the transformation by the metasurface of the photon creation and annihilation operators from the input,  and , where *p* = {*H*, *V*} is the polarization state, to each of the output diffraction orders,  and . This can be expressed through the linear transfer matrix elements as
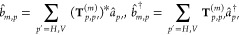
3

We target the tomography of quantum polarization-entangled states with a fixed photon number *N* at the input, which is a common practical task,^17,30−32^ considering the simplest type of click detectors that cannot resolve the number of arriving photons and cannot distinguish the photon polarization state. Notably, the state characterization can also be generalized to the regime when the maximum photon number is known using the approach of ref (33). If no more than one photon arrives at such a detector positioned at the diffraction order *m*, then its response is governed by the following POVM operator^34^
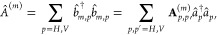
4where using eqs 2 and 3 we calculate the matrix expression
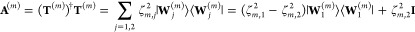
5

We see that this is the sum of a polarization projection operator and a polarization-insensitive detection. The presence of the latter term is a consequence of the partial-polarizer transformation at each of the diffraction orders. Although conventional polarimetry requires near-perfect polarizers (i.e., ζ_*m*,2_ = 0), we apply the POVM formalism that enables unique and accurate quantum state reconstruction in the regime of ζ_*m*,2_ > 0. Beyond metasurfaces, we expect that the formulated approach can also be used to enhance polarization measurements using nanowire detectors,^35^ overcoming the limitations due to relatively low polarization extinction ratios.^36^

After determining the detection operators, we find the probabilities of the simultaneous detection of *N* photons by a combination of *N* detectors at the diffraction orders *m*_1_, *m*_2_, ..., *m*_*N*_, when there is exactly one photon at each detector. Notably, if more than one photon arrives at a particular detector, then the total number of coincidences measured across all detectors will be less than *N* and thus excluded from the analysis. The *N*-detector correlations can be calculated as
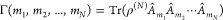
6where ρ^(*N*)^ is an input density matrix. Then, we follow an established procedure^9,37^ to enumerate with index *q* all the possible *N* combinations of *M* detectors (*m*_1_, *m*_2_, ..., *m*_*N*_), and rewrite eq 6 in an equivalent form
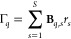
7where *r*_*s*_ is the independent real and imaginary parts of the input density matrix defined according to the procedure in ref (32), *S* = (*N* + 3)!/(3!*N*!), *q* = 1, ..., *Q*, *Q* = *M*!/(*N*!(*M* – *N*)!), and *M* are the total number of detected diffraction orders. The matrix elements **B**_*p*,*s*_ depend on the transfer matrix elements, and more specifically on the vectors **W**_*j*_^(*m*)^ and singular values ζ_*m*,*p*_ according to the form of eq 5.

We can then reconstruct an input state from the correlation measurements by performing a pseudoinversion of eq 7, provided the number of different correlations matches or exceeds the number of unknowns, *Q* ≥ *S*, which is satisfied when
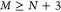
8

Importantly, in addition to the necessary condition in eq 8, it is essential that reconstruction results are robust in the presence of experimental errors in the correlation measurements.^38^ This can be expressed as a requirement to minimize the condition number κ of matrix **B**, defined as a ratio of its largest and smallest singular values.^9,39,40^ The condition number of an equation characterizes the worst-case error in the output for a given error in the set of input parameters. In particular, the condition number of a linear (eq 7) characterizes the accuracy of the reconstruction against errors in the observations.

## Experimental Methods

### Optimized Metagrating Design

**Figure 2:**
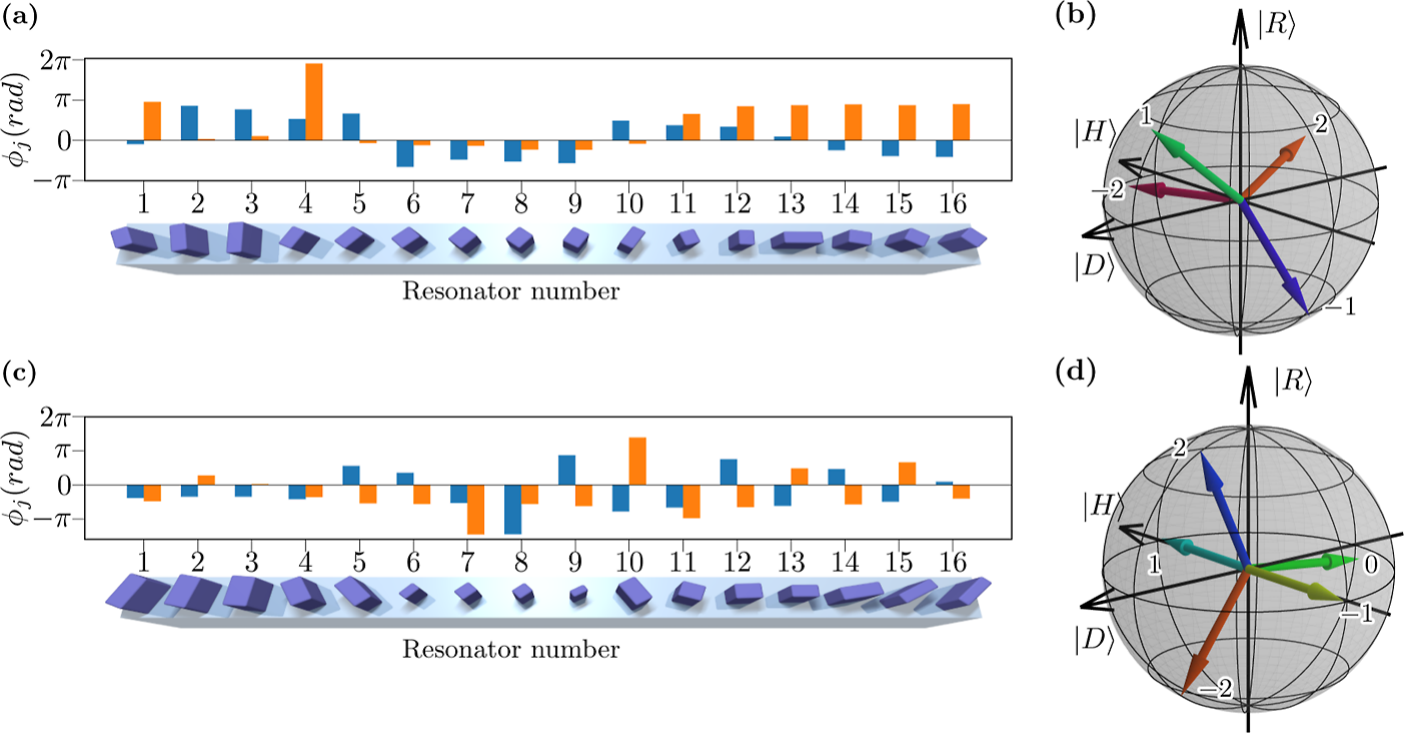
Design of metagratings for (a,b) one-photon or classical and (c,d) two-photon polarimetry. (a,c) Bottom—rendering of metagratings made of amorphous silicon on a glass substrate, illustrating different shapes and orientations of rectangular nanopillars. Top—the phase retardances along the two principal axes of nanopillars shown with blue and orange colors. (b,d) Corresponding Poincaré sphere representation of partial polarizer transformations at different diffraction orders as labeled.

To implement the multiple polarization transformations required to implement the proposed polarimetry scheme, we leverage the flexibility of designing metasurfaces as follows. First, we defined a metagrating consisting of *L* rectangular nanoresonators arranged into a periodic supercell, as shown in Figure 2a,c. A transfer matrix of each nanoresonator can be represented in the Jones formalism^41^ as
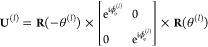
9where *l* is the resonator number, ϕ_o_^(*l*)^ and ϕ_e_^(*l*)^ are the phase shifts imposed by the resonator along the ordinary and extraordinary axes, and **R**(θ^(*l*)^) is a two-by-two rotation matrix by angle θ^(*l*)^. Then, we calculate the transfer matrices of the metasurface at the output diffraction orders using the Fourier transform
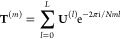
10

We calculate these matrices numerically and then use eqs 4–7 to determine the corresponding condition number κ, defined as the ratio of the maximum and minimum singular value decomposition values of matrix **B** appearing in eq 7. By minimizing the condition number via a gradient-descent algorithm, we can thus obtain a highly robust design. This optimization produces a set of phase parameters corresponding to each pixel of the unit cell, consisting of phase shifts along the ordinary and extraordinary axes, as well as the orientation angles.

We present the optimized metagrating designs for one- and two-photon cases in Figure 2a,c for the minimum required number of outputs according to eq 8, *M* = 4 and *M* = 5, respectively. The numerical values of the nanoresonator parameters are presented in the Supporting Information, Section S1. In these examples, we chose to consider an array of *L* = 16 nanoresonators, but it should be observed that this choice is neither unique nor constrained, and different numbers can be selected depending on the required angular diversion of the diffraction orders after the metasurface.

As implied by eq 5, vectors **W**_*j*_^(*m*)^ are the basis states of the polarization measurement. After converting them to a Stokes basis, we plotted them on a Poincaré sphere for convenient visualization of the basis states at different diffraction orders for a given metasurface design, as shown in Figure 2b,d. We define the vector lengths as
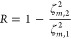
11where ζ_*m*,2_^2^ and ζ_*m*,1_^2^ are by definition proportional to the minimum and maximum powers transmitted to the relevant diffraction order *m* across a set of all possible polarization states under the same input power. We note that *R* = 1 corresponds to a fully polarized output, as would be required for projective measurements. We observe that *R* < 1 for several outputs of our optimized metagratings, meaning that they act as partial polarizers with the finite extinction ratio (1 – *R*)^−1^. Nevertheless, this allows for the efficient reconstruction of the input polarization states.

Indeed, the corresponding inverse condition numbers, calculated for the metagratings that are numerically optimized to maximize their values, are around 0.37 and 0.21 in the spectral region of interest for single- and two-photon states. The single-photon value is comparable to the theoretically best maximum^18^ of . While the best possible value for two-photon state reconstruction with click detectors, which do not resolve the events of both photons exiting from the same port, is not known analytically, previous numerical optimizations of integrated waveguide circuits^30,37^ reported values up to ∼0.25, again close to the two-photon result above in our current study.

### Robustness to Fabrication Errors

Based on the POVM formulation of the design, strong robustness against fabrication errors is expected of the metasurface since accurate reconstruction is possible even if the polarization extinction ratios at individual outputs are affected. We have carried out numerical simulations to demonstrate this, considering as representative examples the metasurface designs for single- and two-photon polarimetry presented above.

Under realistic fabrication scenarios, the most common deviations from the analytical design pertain to the overall sizes of the nanoresonators. These were modeled as variance in the phase shifts along the ordinary and extraordinary axes of the individual nanopixels, corresponding to the decomposition shown in eq 9, and thus, random errors up to Δϕ were added to each nanopixel, and then the transmission of the altered metasurface structure was calculated via eq 10. From this result, we computed for the perturbed metasurface the overall inverse condition numbers 1/κ and diffraction efficiency η_min_.

**Figure 3:**
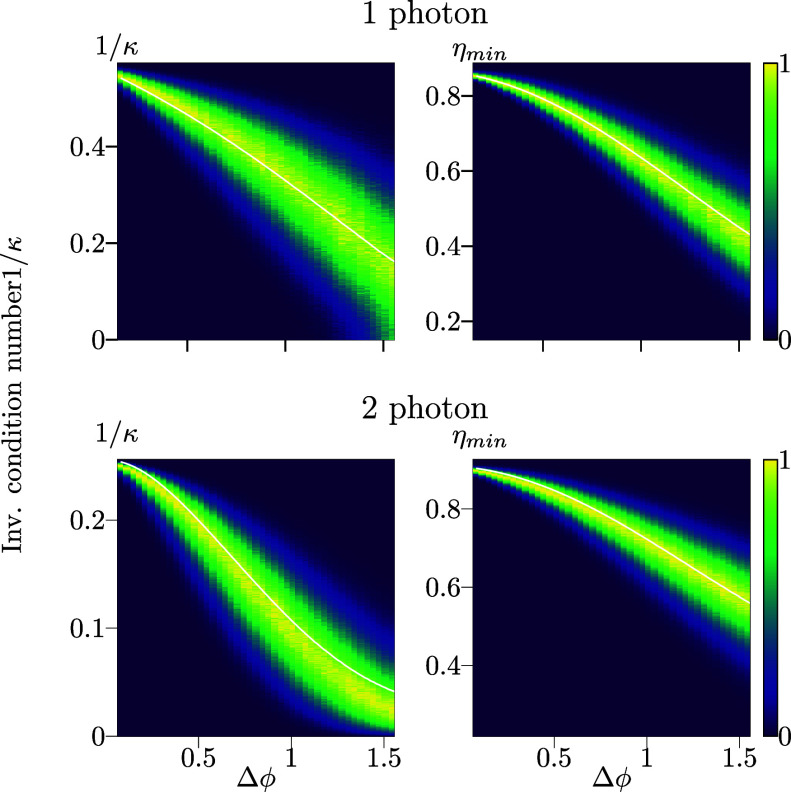
Simulated effects of random phase errors, up to a maximum of Δϕ, applied to each of the nanoresonators in the metagratings optimized for one-photon and two-photon polarimetry, as illustrated in Figure 2a,c, respectively. Shown are the probability densities, normalized to a maximum of unity at each Δϕ, for the inverse condition numbers 1/κ and diffraction efficiencies η_min_. White lines indicate the average values.

The diffraction efficiency is defined as the minimum fraction of the total input power that is diffracted to the chosen orders used in computing the corresponding inverse condition number, specifically to the (±1, ±2) orders in the one-photon case and (0, ±1, ±2) orders in the two-photon case. By repeating such simulations over 10^5^ times for each value of Δϕ, we estimated the degradation of performance vs the degree of random errors, as summarized in Figure 3. The errors trialed range up to an extreme case of Δϕ = π/2, which far exceeds the possible effects of the nanofabrication errors. We also note that in the two-photon case, the zero-order diffraction was included in the computation of both 1/κ and η_min_, whereas the single-photon case excludes the zero order. The additional basis state provided by zero-order diffraction was found to slightly increase the achievable diffraction efficiency in the two-photon case.

We find that for realistic levels of errors below 10^–1^, both the inverse condition number and the diffraction efficiencies do not drop significantly, as shown in Figure 3. However, as one might expect, the two-photon design is more sensitive to errors than the single-photon case, owing to the necessary consideration of additional diffraction orders to fully resolve multiple photons.

## Experimental Results

### Metasurface Patterns: Design and Fabrication

We first determine the physical dimensions of nanoresonators, realizing the required phase delays, such as those summarized in Supporting Information, Section S1, using an established procedure.^9,41^ Specifically, we perform a sweep of the length and width parameters of cuboidal pixels, while keeping fixed the metagrating period, height, and refractive indices, and calculate the transmission using a numerical technique known as rigorous coupled-wave analysis.^42−44^ Thereby, we produce a lookup table from which suitable designs could be simply selected. Thereby, one can design a metasurface for operating at the desired spectral regions.

As a demonstration, we developed a metasurface for operation at the telecommunication band, around the 1550 nm wavelength. As a platform, we chose a dielectric metasurface made of amorphous silicon on a glass substrate, leveraging the high transmissivity to ensure a high efficiency of operation. The height and period used were 832 and 800 nm, respectively, the former corresponding to measurements of deposited silicon that would be used to fabricate the metasurface. The physical parameters of the metasurface were thus designed by selecting pixels that fit the desired phase parameters, up to an arbitrary global phase. The combined metasurface structure designed was then simulated using a commercial electrodynamics solver, CST Studio, as a final optimization pass and to check the influence of optical couplings between adjacent pixels that are not accounted for in the design of individual nanoresonators. This optimization pass did not alter the design significantly, changing the design parameters by <2%.

The metasurfaces were fabricated from an 832 nm-thick amorphous silicon layer prepared at the ANU node of the Australian National Fabrication Facility (ANFF) using plasma-enhanced chemical vapor deposition (PECVD) on a glass substrate. It was subsequently etched at the University of Jena by using electron beam lithography (EBL) and inductively coupled plasma etching. Slight variants in the metasurfaces were prepared by intentionally varying the EBL exposure times, allowing for a selection of best-case fabrication outcomes.

## Results and Discussion

After fabrication, we measured the transmission of classical light through the metasurface. This enables the characterization of the metasurface transformation from the input to distinct outputs, which can then be used for the reconstruction of multiphoton states. We use a classical-quantum analogy^29^ since both the single-photon (for *N* = 1) counts at the outputs and the classical output intensities can be described by eqs 6, 7. Here, the density matrix can be conveniently related to the Stokes parameters *S⃗*(17,45,46)
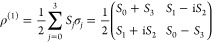
12where σ_*j*_ is the Pauli matrices. For convenience, we choose the independent parameters in the input density matrix as . Then, the matrix **B** in eq 7 has the meaning of an instrument matrix, since
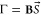
13determines the intensities at the output diffraction orders for classical light with the input Stokes parameters **S**. Specifically, at the output number *m*
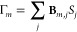
14

On the other hand, using eq 6 at *N* = 1, the output intensities can be expressed as
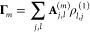
15

We then obtain
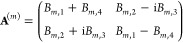
16

In this way, the classical instrument matrix **B** can be used to determine all matrices **A**^(*m*)^ and thereby allow the subsequent reconstruction of arbitrary multiphoton states.

**Figure 4:**
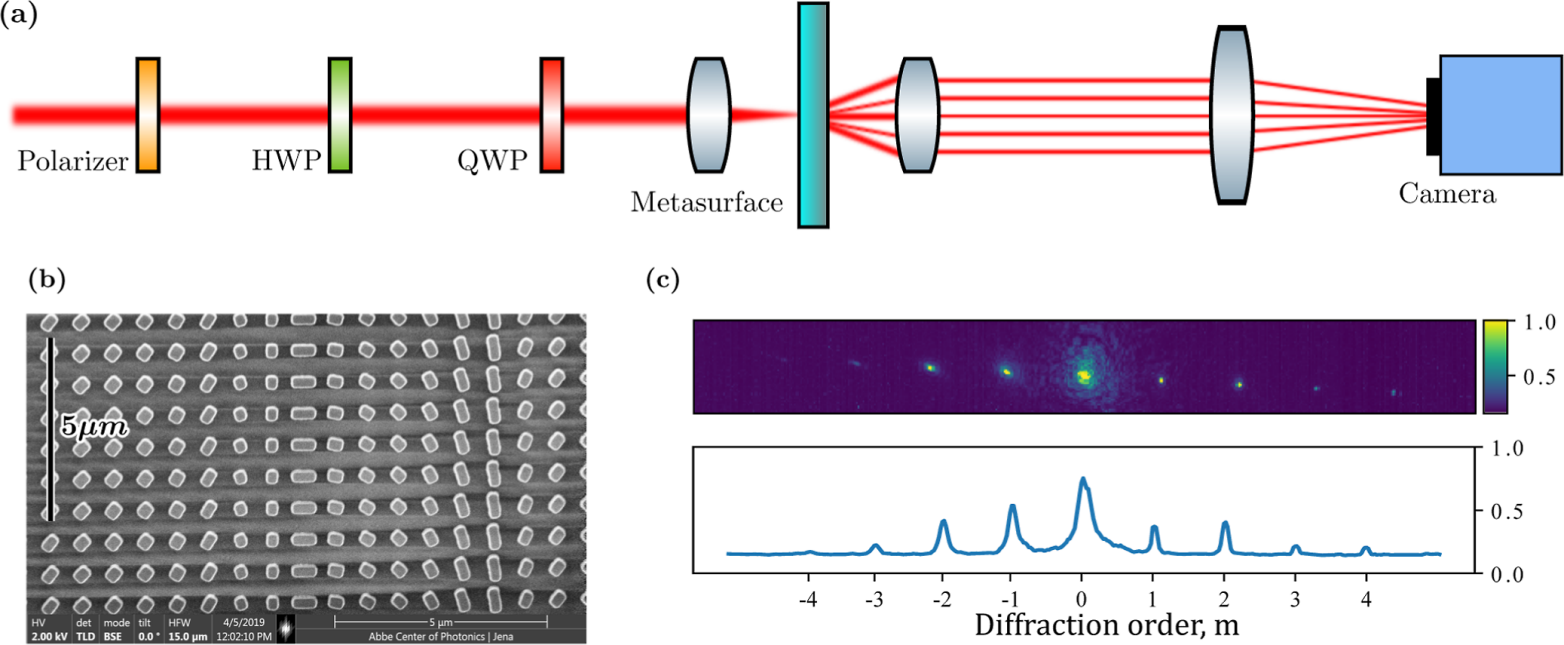
(a) Schematic of the experimental setup used to classically characterize the metasurface. Input states were prepared from a variable-wavelength infrared laser using a fixed polarizer and motorized half- and quarter-waveplates before being collimated on the metasurface by lenses. Output diffraction order intensities were collected using a CCD camera. (b) Scanning electron microscope image of the metasurface, fabricated as 832 nm amorphous silicon on glass via EBL. (c) Representative readings as taken using the camera and the processed intensity plot obtained by slicing across the diffraction orders. Intensities were normalized to 1.

The experimental setup is schematically illustrated in Figure 4a. Using a half-wave plate, quarter-wave plate, and fixed polarizer, polarization states were prepared from a variable-wavelength laser operating in the 1500–1575 nm telecommunications bandwidth. The prepared polarization state was then focused to a spotsize of approximately 15 μm normally incident on the metasurface. The diffraction orders were then collected using an objective lens with a high numerical aperture and imaged onto an infrared CCD camera using a convex lens. As a separate measurement, the camera was replaced with a calibrated power meter in order to determine the total power that was transmitted through the metasurface.

We measured the intensities of the diffraction orders over varying input polarization orders by capturing images on a camera, as shown in Figure 4c. First, the diffraction order spot locations were determined, and then the individual intensities were extracted by integrating over the area of each diffraction spot.

We performed measurements for a calibration set of 360 distinct input polarization states and calculated instrument matrix **B** for diffraction orders (±2, ±1) by fitting its parameters according to eq 13 using the known input states and measured output powers. We note that each diffraction output intensity is defined by a row of the matrix **B** that contains four elements, and therefore four or more calibration measurements are needed to determine uniquely all the **B** elements. We intentionally used a large size of the calibration set to reduce the effect of random noise in individual measurements through the averaging.

**Figure 5:**
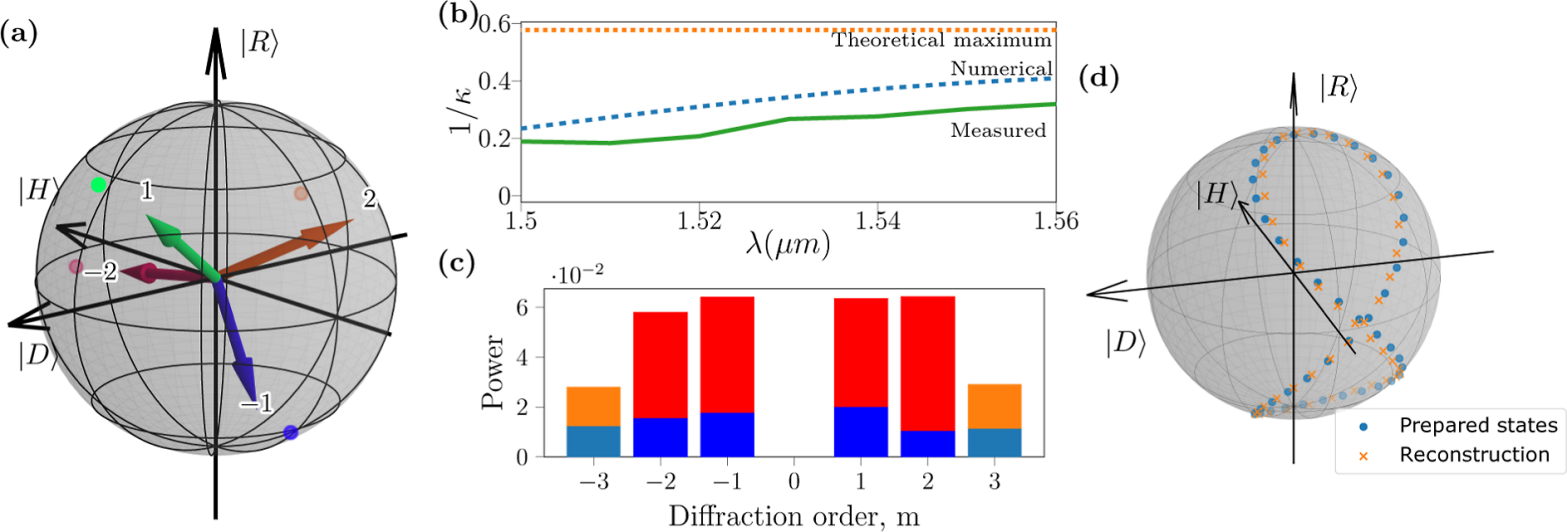
(a) Poincaré sphere representation of the basis states as calculated from the (±2, ±1) diffraction orders of the fabricated metasurface. These are, as predicted, partially polarized states that deviate from the original numerical design, shown as correspondingly colored dots on the Poincaré sphere. (b) Experimentally characterized inverse condition numbers of the metasurface across a wavelength range are plotted using the solid green line. Blue, dashed line represents the numerical result through CST Studio simulations of the optimal metasurface design, and the orange, dotted line represents the theoretical maximum. (c) Minimum (blue) and maximum (red) power directed to each diffraction order, as determined by the experiment. Diffraction orders ±3 were measured but not utilized in the calculations, and are shown for comparison. (d) Poincaré sphere showing a comparison of input states versus the reconstructed states using the experimentally characterized metasurface.

We compute the polarization bases of the measured instrument matrix and plot a representative example in Figure 5a, corresponding to a wavelength of 1560 nm. Here, we see that the metasurface operation has deviated from the designed values, as shown in Figure 2a, which may be ascribed to fabrication errors. Nevertheless, these do not prevent the polarization characterization, as we discuss below.

We use the measured instrument matrix to compute the inverse condition number dependence on the wavelengths; see Figure 5b. Importantly, it is only slightly lower (by up to 15%) than the numerical design values indicated with the dashed line across the whole target operating bandwidth. For comparison, the dotted line marks the theoretical maximum of .^18^ Such experimentally achieved performance is close to the value of 1/2.08 ≃ 0.48 for an interleaved metasurface,^9^ yet it provides a fundamentally better beam quality without distortions in the vertical direction.

We use the experimental data to visualize the minimum and maximum powers across all possible input polarization states and normalized them to the input power at the selected output ports, as shown in Figure 5c. We note that the ratios of these minimum to maximum powers define the polarization extinction, which appears to be on the order of 10^–1^. Despite such a low extinction, an accurate state reconstruction is still possible using the developed approach based on the framework of generalized quantum measurements.

We demonstrate the reconstruction of the input states from the measured powers at the selected diffraction orders (±2, ±1) after the metasurface. From experimental data, a random set of angles for the half- and quarter-waveplates was chosen. From these angles and taking into account that the first polarizer was positioned vertically, we thus define the input polarization states ρ_in_^(1)^ before incidence on the metasurface. We also independently reconstruct the input states from the measured intensities  and the experimentally characterized instrument matrix **B**, as
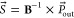
17and then determine the input density matrix using eq 12. We plot the prepared and reconstructed states on the same Poincaré sphere for comparison, as shown in Figure 5d.

In this plot, we show 44 data points, of which only 4 overlap with the calibration data set, thereby providing a valid test of the reconstruction accuracy for unknown input states. We quantify the difference between the normalized input  and reconstructed  Stokes vector as , such that δ = 0 only if the vectors are the same, up to a total intensity. Then, we find that for the states in Figure 5d, the error in reconstruction is δ ≤ 2.9%.

Whereas multiphoton quantum experiments are beyond the scope of the current work, we use the experimentally characterized instrument matrix of the metasurface to calculate an inverse condition number for two-photon reconstruction and find that κ^–1^ ≃ 0.15 can be achieved at a wavelength of 1520 nm (see Supporting Information, Section S2 for a wavelength dependence), close to the theoretically optimized value of 0.21.

## Conclusions

We have presented a general approach for the complete measurement of polarization in both quantum multiphoton states and classical light using nanostructured metasurfaces. Our method nontrivially combines the principles of single-shot polarimetry and generalized quantum measurements with the POVM formalism, enabling accurate polarization reconstruction even in the presence of significant fabrication errors or environmental changes, by performing a simple device calibration. Our experimental measurements demonstrate a complete reconstruction of classical polarization states with a maximum error of 2.9% while maintaining a high optical beam quality after the metasurface for efficient detection. We anticipate that our new concept will facilitate diverse quantum and classical applications, from laboratories to satellite imaging systems, benefiting from extremely compact metadevices providing real-time polarimetric measurements.

## Supporting Information Available

Numerically optimized values of phase shifts corresponding to the metasurface and experimentally calculated two-photon inverse condition number (PDF)
Single-shot polarimetry (ZIP)

## References

[ref1] Martinez A. Polarimetry enabled by nanophotonics. Science 2018, 362, 750–751. 10.1126/science.aau7494. 30442792

[ref2] Intaravanne Y.; Chen X. Z. Recent advances in optical metasurfaces for polarization detection and engineered polarization profiles. Nanophotonics 2020, 9, 1003–1014. 10.1515/nanoph-2019-0479.

[ref3] Rubin N. A.; Shi Z. J.; Capasso F. Polarization in diffractive optics and metasurfaces. Adv. Opt. Photon 2021, 13, 836–970. 10.1364/AOP.439986.

[ref4] Pors A.; Nielsen M. G.; Bozhevolnyi S. I. Plasmonic metagratings for simultaneous determination of Stokes parameters. Optica 2015, 2, 716–723. 10.1364/OPTICA.2.000716.

[ref5] Zhang X. Q.; Yang S. M.; Yue W. S.; Xu Q.; Tian C. X.; Zhang X. X.; Plum E.; Zhang S.; Han J. G.; Zhang W. L. Direct polarization measurement using a multiplexed Pancharatnam-Berry metahologram. Optica 2019, 6, 1190–1198. 10.1364/OPTICA.6.001190.

[ref6] Arbabi E.; Kamali S. M.; Arbabi A.; Faraon A. Full-Stokes Imaging Polarimetry Using Dielectric Metasurfaces. ACS Photonics 2018, 5, 3132–3140. 10.1021/acsphotonics.8b00362.

[ref7] Wei S. W.; Yang Z. Y.; Zhao M. Design of ultracompact polarimeters based on dielectric metasurfaces. Opt. Lett. 2017, 42, 1580–1583. 10.1364/OL.42.001580. 28409803

[ref8] Yang Z. Y.; Wang Z. K.; Wang Y. X.; Feng X.; Zhao M.; Wan Z. J.; Zhu L. Q.; Liu J.; Huang Y.; Xia J. S.; Wegener M. Generalized Hartmann-Shack array of dielectric metalens sub-arrays for polarimetric beam profiling. Nat. Commun. 2018, 9, 4607 10.1038/s41467-018-07056-6. 30389933 PMC6214988

[ref9] Wang K.; Titchener J. G.; Kruk S. S.; Xu L.; Chung H. P.; Parry M.; Kravchenko I. I.; Chen Y. H.; Solntsev A. S.; Kivshar Y. S.; Neshev D. N.; Sukhorukov A. A. Quantum metasurface for multiphoton interference and state reconstruction. Science 2018, 361, 1104–1108. 10.1126/science.aat8196. 30213910

[ref10] Chekhova M.; Banzer P. Polarization of Light in Classical, Quantum, and Nonlinear Optics; De Gruyter: Berlin, 2021.

[ref11] Rubin N. A.; D’Aversa G.; Chevalier P.; Shi Z. J.; Chen W. T.; Capasso F. Matrix Fourier optics enables a compact full-Stokes polarization camera. Science 2019, 365, eaax1839 10.1126/science.aax1839. 31273096

[ref12] Rubin N. A.; Chevalier P.; Juhl M.; Tamagnone M.; Chipman R.; Capasso F. Imaging polarimetry through metasurface polarization gratings. Opt. Express 2022, 30, 9389–9412. 10.1364/OE.450941. 35299368

[ref13] Chen W. T.; Torok P.; Foreman M. R.; Liao C. Y.; Tsai W. Y.; Wu P. R.; Tsai D. P. Integrated plasmonic metasurfaces for spectropolarimetry. Nanotechnology 2016, 27, 224002 10.1088/0957-4484/27/22/224002. 27114455

[ref14] Ding F.; Pors A.; Chen Y. T.; Zenin V. A.; Bozhevolnyi S. I. Beam-Size-Invariant Spectropolarimeters Using Gap-Plasmon Metasurfaces. ACS Photonics 2017, 4, 943–949. 10.1021/acsphotonics.6b01046.

[ref15] Sun T.; Hu J. P.; Zhu X. J.; Xu F.; Wang C. H. Broadband Single-Chip Full Stokes Polarization-Spectral Imaging Based on All-Dielectric Spatial Multiplexing Metalens. Laser Photon. Rev. 2022, 16, 2100650 10.1002/lpor.202100650.

[ref16] Camayd-Munoz P.; Ballew C.; Roberts G.; Faraon A. Multifunctional volumetric meta-optics for color and polarization image sensors. Optica 2020, 7, 280–283. 10.1364/OPTICA.384228.

[ref17] James D. F. V.; Kwiat P. G.; Munro W. J.; White A. G. Measurement of qubits. Phys. Rev. A 2001, 64, 052312 10.1103/PhysRevA.64.052312.

[ref18] Foreman M. R.; Favaro A.; Aiello A. Optimal Frames for Polarization State Reconstruction. Phys. Rev. Lett. 2015, 115, 263901 10.1103/PhysRevLett.115.263901. 26764991

[ref19] Pittman T. B.; Shih Y. H.; Strekalov D. V.; Sergienko A. V. Optical imaging by means of two-photon quantum entanglement. Phys. Rev. A 1995, 52, R3429–R3432. 10.1103/PhysRevA.52.R3429. 9912767

[ref20] Altuzarra C.; Lyons A.; Yuan G. H.; Simpson C.; Roger T.; Ben-Benjamin J. S.; Faccio D. Imaging of polarization-sensitive metasurfaces with quantum entanglement. Phys. Rev. A 2019, 99, 020101 10.1103/PhysRevA.99.020101.

[ref21] Vega A.; Pertsch T.; Setzpfandt F.; Sukhorukov A. A. Metasurface-Assisted Quantum Ghost Discrimination of Polarization Objects. Phys. Rev. Appl. 2021, 16, 064032 10.1103/PhysRevApplied.16.064032.

[ref22] Brandt H. E. Positive operator valued measure in quantum information processing. Am. J. Phys. 1999, 67, 434–439. 10.1119/1.19280.

[ref23] Hamieh S.; Kobes R.; Zaraket H. Positive-operator-valued measure optimization of classical correlations. Phys. Rev. A 2004, 70, 052325 10.1103/PhysRevA.70.052325.

[ref24] Renes J. M.; Blume-Kohout R.; Scott A. J.; Caves C. M. Symmetric informationally complete quantum measurements. J. Math. Phys. 2004, 45, 2171–2180. 10.1063/1.1737053.

[ref25] Ziman M. Process positive-operator-valued measure: A mathematical framework for the description of process tomography experiments. Phys. Rev. A 2008, 77, 062112 10.1103/PhysRevA.77.062112.

[ref26] Shapiro J. H. Continuous positive-operator-valued measurement of photon polarization. Phys. Rev. A 2008, 77, 052330 10.1103/PhysRevA.77.052330.

[ref27] Al Khafaji M. A.; Cisowski C. M.; Jimbrown H.; Croke S.; Padua S.; Franke-Arnold S. Single-shot characterization of vector beams by generalized measurements. Opt. Express 2022, 30, 22396 10.1364/OE.458352. 36224938

[ref28] Martinez D.; Gomez E. S.; Carine J.; Pereira L.; Delgado A.; Walborn S. P.; Tavakoli A.; Lima G. Certification of a non-projective qudit measurement using multiport beamsplitters. Nat. Phys. 2022, 19, 190–197. 10.1038/s41567-022-01845-z.

[ref29] Barnett S. M. On single-photon and classical interference. Phys. Scr. 2022, 97, 114004 10.1088/1402-4896/ac971a.

[ref30] Titchener J. G.; Solntsev A. S.; Sukhorukov A. A. Two-photon tomography using on-chip quantum walks. Opt. Lett. 2016, 41, 4079–4082. 10.1364/OL.41.004079. 27607977

[ref31] Oren D.; Mutzafi M.; Eldar Y. C.; Segev M. Quantum state tomography with a single measurement setup. Optica 2017, 4, 993–999. 10.1364/OPTICA.4.000993.

[ref32] Titchener J. G.; Grafe M.; Heilmann R.; Solntsev A. S.; Szameit A.; Sukhorukov A. A. Scalable on-chip quantum state tomography. npj Quant. Inform. 2018, 4, 19 10.1038/s41534-018-0063-5.

[ref33] Bayraktar O.; Swillo M.; Canalias C.; Bjork G. Quantum-polarization state tomography. Phys. Rev. A 2016, 94, 020105 10.1103/PhysRevA.94.020105.

[ref34] Kok P.; Lovett B. W. Introduction to Optical Quantum Information Processing; Cambridge University Press: Cambridge, 2010.

[ref35] Zhu Y.; Raj V.; Li Z. Y.; Tan H. H.; Jagadish C.; Fu L. Self-Powered InP Nanowire Photodetector for Single-Photon Level Detection at Room Temperature. Adv. Mater. 2021, 33, 2105729 10.1002/adma.202105729. 34622479

[ref36] Park H.; Crozier K. B. Elliptical silicon nanowire photodetectors for polarization-resolved imaging. Opt. Express 2015, 23, 7209–7216. 10.1364/OE.23.007209. 25837065

[ref37] Wang K.; Suchkov S. V.; Titchener J. G.; Szameit A.; Sukhorukov A. A. Inline detection and reconstruction of multiphoton quantum states. Optica 2019, 6, 41–44. 10.1364/OPTICA.6.000041.

[ref38] Filippov S. N.; Man’ko V. I. Inverse spin-s portrait and representation of qudit states by single probability vectors. J. Russ. Laser Res. 2010, 31, 32–54. 10.1007/s10946-010-9122-x.

[ref39] Bogdanov Y. I.; Kulik S. P.; Moreva E. V.; Tikhonov I. V.; Gavrichenko A. K. Optimization of a quantum tomography protocol for polarization qubits. JETP Lett. 2010, 91, 686–692. 10.1134/S0021364010120143.

[ref40] Miranowicz A.; Bartkiewicz K.; Perina J.; Koashi M.; Imoto N.; Nori F. Optimal two-qubit tomography based on local and global measurements: Maximal robustness against errors as described by condition numbers. Phys. Rev. A 2014, 90, 062123 10.1103/PhysRevA.90.062123.

[ref41] Arbabi A.; Horie Y.; Bagheri M.; Faraon A. Dielectric metasurfaces for complete control of phase and polarization with subwavelength spatial resolution and high transmission. Nat. Nanotechnol. 2015, 10, 937–943. 10.1038/nnano.2015.186. 26322944

[ref42] Lalanne P. Improved formulation of the coupled-wave method for two-dimensional gratings. J. Opt. Soc. Am. A 1997, 14, 1592–1598. 10.1364/JOSAA.14.001592.

[ref43] Lalanne P.; Morris G. M. Highly improved convergence of the coupled-wave method for TM polarization. J. Opt. Soc. Am. A 1996, 13, 779–784. 10.1364/JOSAA.13.000779.

[ref44] Hugonin J. P., Lalanne P. Reticolo software for grating analysis. arXiv preprint arXiv:2101.00901. 2021, (accessed Mar 01, 2020).

[ref45] Fano U. Remarks on the classical and quantum-mechanical treatment of partial polarization. J. Opt. Soc. Am. 1949, 39, 859–863. 10.1364/JOSA.39.000859.

[ref46] Goldberg A. Z.; De La Hoz P.; Bjork G.; Klimov A. B.; Grassl M.; Leuchs G.; Sánchez-Soto L. L. Quantum concepts in optical polarization. Adv. Opt. Photon 2021, 13, 1–73. 10.1364/AOP.404175.

