# Supplementary material for: Robust Classical and Quantum Polarimetry with a Single Nanostructured Metagrating
Source: ACS Photonics. 2024 Feb 15;11(3):1060–7. doi: 10.1021/acsphotonics.3c01287 (PMC10958599; doi:10.1021/acsphotonics.3c01287)
Supplement: Supplementary file 1 — ph3c01287_si_001.pdf [file ph3c01287_si_001.pdf]

Supplementary Information: Robust Classical and Quantum Polarimetry with a Single Nanostructured Metagrating

Shaun Lung,*,†,‡ Kai Wang,¶ Nicolas R.H. Pedersen,† Frank Setzpfandt,†,§ and Andrey A. Sukhorukov‡

†*Abbe Center of Photonics, Friedrich-Schiller Universität, Albert-Einstein-Straße 15, 07745 Jena, Germany*

‡*ARC Centre of Excellence for Transformative Meta-Optical Systems (TMOS), Department of Electronic Materials Engineering, Research School of Physics, The Australian National University, Canberra, ACT 2600, Australia*

¶*Department of Physics, McGill University, 3600 rue University, Montreal, Quebec H3A 2T8, Canada*

§*Fraunhofer Institute for Applied Optics and Precision Engineering, 07745 Jena, Germany*

E-mail: shaun.lung@uni-jena.de

Abstract

This Supporting Information contains 2 sections, 1 table and 1 figure, providing extra details on our work.

(February 7, 2024)

S1 Optimized metasurface parameters

Table S1 contains the numerically optimized values of phase shifts along the x and y directions as well as the angle of rotations of each nanoresonator, corresponding to the metasurface renders shown in Figs. 2(a,c).

Table S1: Phases and rotations in radians of nanoresonators forming metasurfaces optimized for one- and two-photon polarimetry.

Resonator no.	One-photon polarimetry			Two-photon polarimetry		
	ϕ_x	ϕ_y	θ	ϕ_x	ϕ_y	θ
1	-0.29	-0.15	0.43	-1.19	-1.5	0.72
2	-0.45	0.11	-1.34	-1.08	0.85	0.94
3	-0.73	0.33	-1.48	-1.09	0.078	-0.092
4	-1.48	-0.30	1.1	-1.31	-1.12	-0.28
5	-1.06	-0.22	0.86	-1.39	1.44	0.76
6	1.08	-0.38	0.75	1.11	1.38	0.53
7	-1.51	-0.42	0.83	1.47	-1.42	-1.29
8	1.52	-0.71	0.86	-1.4	1.39	0.128
9	1.36	-0.72	1.01	-0.40	1.19	-0.0016
10	1.54	-0.26	1.0	0.7	1.22	-0.362
11	1.18	-1.13	-1.25	1.06	0.072	0.26
12	1.05	-0.48	1.52	-0.77	1.11	1.42
13	0.29	-0.41	1.56	1.2	1.52	-0.29
14	-0.76	-0.34	1.37	1.46	1.34	1.17
15	-1.22	-0.41	1.02	-1.55	-1.07	-0.72
16	-1.29	-0.33	0.77	0.288	-1.25	0.66

S2 2-photon condition number

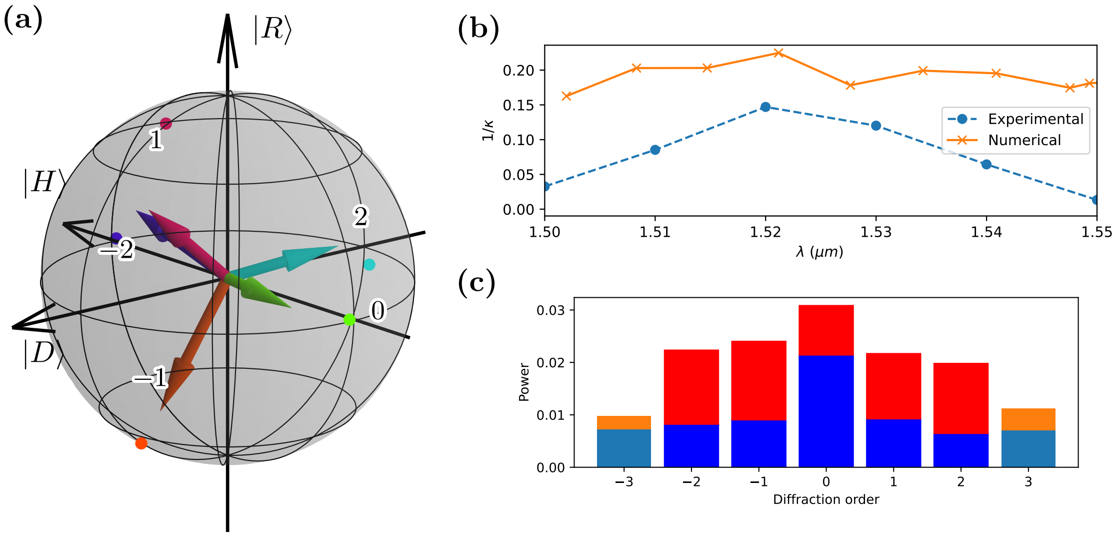

Figure S1: (a) Poincaré sphere representation of the basis states as calculated from the $(\pm 2, \pm 1, 0)$ diffraction orders of the fabricated metasurface. (b) The two-photon state reconstruction condition numbers vs. the wavelength determined from the experimentally characterized metasurface instrument matrix (green line) and from the finite-difference numerical simulations with CST studio (blue line). (c) The minimum (blue) and maximum (red) power directed to each diffraction order, as determined by experiment.
