# Supplementary figures and images for: Robust Classical and Quantum Polarimetry with a Single Nanostructured Metagrating
Source: ACS Photonics. 2024 Feb 15;11(3):1060–7. doi: 10.1021/acsphotonics.3c01287 (PMC10958599; doi:10.1021/acsphotonics.3c01287)

(a)

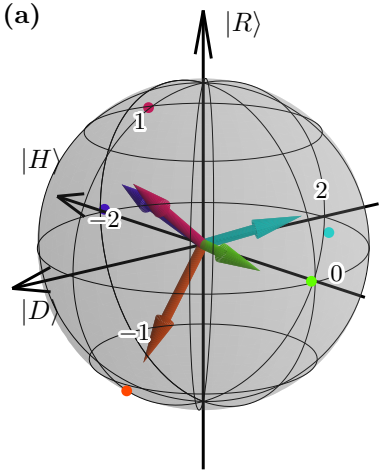

(b)

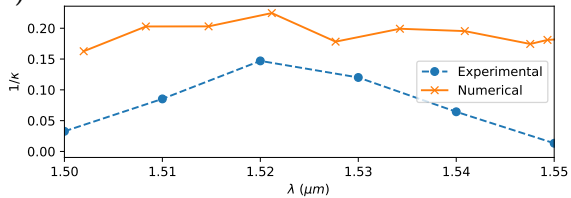

(c)

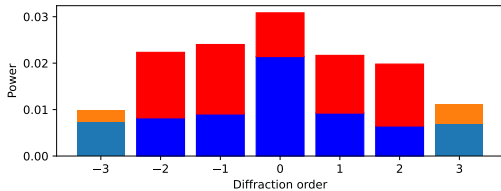

Supplement: Supplementary file 2 — ph3c01287_si_002.zip [file ph3c01287_si_002.zip › figures/sup.pdf]
